# Using ontologies to facilitate healthcare process mining and analysis

**DOI:** 10.1007/s10844-025-00942-8

**Published:** 2025-05-29

**Authors:** Owen P Dwyer, Lara Chammas, Emanuel Sallinger, Jim Davies

**Affiliations:** 1 University of Oxford, Oxford, UK; 2 TU Wien, Vienna, Austria

**Keywords:** Process mining, Ontologies, Electronic health records

## Abstract

Healthcare organisations collect detailed data on the care that they deliver. This data can be used to identify issues, including deviations from care standards and recommendations, and opportunities for improvement; it can be used also to support the development of new technologies and treatments. The volume and complexity of the data means that automated techniques such as process mining are needed to support the extraction and analysis of relevant information. This paper explains how the ontological information held in clinical terminologies can be used to facilitate process extraction and analysis, by connecting and aggregating clinical events through the classification of diagnoses made and treatments performed. The approach is demonstrated through application to data collected on care delivered to patients with cancer in a major hospital. The results are compared with those obtained from benchmark datasets using approaches in which connections and aggregations are proposed and curated by domain experts. This comparison highlights the potential, and the shortcomings, of ontology-based extraction and analysis in healthcare process mining.

**Supplementary Information:**

The online version contains supplementary material available at 10.1007/s10844-025-00942-8.

## Introduction

A clinical pathway describes the sequence of events that patients are expected to experience over the course of their journey through the healthcare system. The relationship between these idealised models and the real journeys experienced by patients is an area of significant research interest, and the increasing availability of large databases of electronic health records (EHR) means that researchers now have access to the data needed to investigate these pathways at scale.

Process mining (PM) is a popular methodology for the analysis of data generated by business processes, and work is underway to apply and extend it within the healthcare domain (Munoz-Gama et al., 2022). However, healthcare processes come with a number of challenges: they are highly dynamic, changing as new interventions and technologies are introduced; they are highly complex, involving large amounts of data and unpredictable events; they are increasingly multidisciplinary, requiring communication and coordination between multiple specialists; and they are ad hoc, modified and interpreted according to individual preferences and professional judgement (Rebuge and Ferreira, 2012). The resulting datasets are characterised by incomplete and noisy signals, high levels of process variation, and multitudes of exceptional behaviours that should ideally be captured rather than disregarded.

Given the complex state of raw healthcare data, preparing it for analysis is a significant and time-consuming undertaking; the many modelling decisions and assumptions that need to be made can have a significant impact on the eventual results. Typically, when determining which clinical events to include or exclude for analysis, researchers need to either manually curate a list of events of interest, or use statistical measures to distinguish between common events and outliers. Human curation of events of interest—ideally with the involvement of domain experts—ensures a high degree of reliability, but it is time-consuming and becomes increasingly challenging in a healthcare scenario, where the possible events in a healthcare record can number in the hundreds or thousands. A statistical approach, for example only looking at the most common events, can be an easy way to simplify data for analysis, but it risks ignoring rare but significant exceptions, or conversely including very common events that are wholly unrelated to the disease of interest.

This paper therefore proposes a third approach to preparing raw healthcare data for process analysis: an ontological approach. Given that healthcare data is typically encoded in standardised terminologies, and that these terminologies are mappable to ontologies rich with semantics, we propose using these ontologies to facilitate the process of event log generation. By specifying domain-informed constraints, the relationships between diseases and events can be deduced, helping to reduce a large dataset of events to a smaller subset of more informative and pertinent ones.

Exploiting structured domain knowledge from ontologies has the potential to bring a number of improvements to the process of event log generation, such as:

- expediting the slow and labour-intensive process of curating lists of relevant terminology concepts (“codelists”)
- allowing codelists to be expressed as a set of intuitive, explainable rules
- providing rules that can be translated into dataset-specific terminologies, enabling reproducibility across healthcare systems and datasets
- promoting reproducibility and code sharing within the research community
- providing an approach available irrespective of a dataset’s sample size

To properly understand the processes used in modern healthcare, PM practice needs to be aligned with and informed by the methods used in epidemiological and healthcare research, to maximise the relevance of results to the healthcare community (Chammas et al., 2024). In this paper, we further this goal by drawing a parallel between event log generation as performed in PM, and codelist curation as used in healthcare research, and investigate the extent to which these processes can be enhanced by structured domain knowledge. The main contributions of this paper are:

- an approach to **extracting** events from raw healthcare data that uses domain knowledge to automatically deduce relationships between diagnoses and procedures to determine relevance.
- an **evaluation** of our proposed approach, by comparing against more traditional approaches, and by demonstrating it on real-world patient data.
- a discussion of **abstracting** events, and how ontological structure might be used to aid in the grouping of events into useful categories for analysis.

The proposed approach addresses two of the key challenges in current healthcare PM as described by Munoz-Gama et al. (2022): we *design a dedicated methodology*, describing a healthcare-specific PM approach that leverages domain knowledge and facilitates comparison across healthcare providers; and *deal with reality* by demonstrating our approach on real-world datasets.

This paper expands on our previously published work (Dwyer et al., 2024), providing a more thorough evaluation against abstraction and extraction tasks, and evaluating in a wider range of scenarios. We first introduce the relevant background material in Section 2, before describing the proposed set of methods in Section 3. The approach is evaluated in Section 4 and discussed in Section 5. Finally, we consider whether it effectively resolves the challenges identified, and explore the potential for future research (Section 6).

## Background

This section introduces key concepts in patient pathways and process mining. We discuss current approaches to filtering healthcare event logs, and—given that we have previously highlighted the benefits of epidemiological methods informing process mining methods, and vice versa (Chammas et al., 2024)—we also examine how the event selection process works in the existing healthcare literature. We also introduce the key terminologies and ontologies used to record healthcare data.

### Patient pathways and process mining

In healthcare, *pathways* are a type of guideline that exist to standardise care for a particular condition or group of patients. They represent a recommended or expected “route” that a patient should take through the healthcare system, spanning identification, diagnosis, treatment and follow-up. Whilst no single accepted definition exists, a pathway is typically viewed as being distinct from a clinical guideline, and serves to translate and tailor high-level treatment recommendations to the circumstances and environment of a specific care provider (Rotter et al., 2019).

The widespread use of pathways in practice prompts a major research question: what do the real-life pathways of patients actually look like? The increasing availability of large-scale data resources from healthcare providers means that we can begin to investigate this on a population scale, but the right methods are required to analyse this data effectively. Process mining (PM) is a family of data science methods that focuses on the discovery, analysis, and improvement of business processes from *event logs*: datasets describing sequences of events (van der Aalst, 2012). The potential benefits of using these methods to investigate patient pathways are significant; for example, analysing treatment processes from two different providers means that we can compare and contrast patterns of treatment (Mans et al., 2008).

However, results have not always been this successful. Despite the great potential of process mining, classic PM techniques often perform poorly on medical data. They are ill-equipped to deal with unstructured processes, and are strongly influenced by noise and incompleteness as well as the sheer number of possible process variants. One of the greatest challenges is the very high level of variability between patients (Munoz-Gama et al., 2022). For PM to be effective, an event log that describes a patient’s treatment history need to be first filtered to only contain events that are relevant to a particular treatment pathway or disease being studied, lest analysis produce meaningless and uninterpretable “spaghetti models”.

### Preparing healthcare data

Before analysis techniques such as PM can be applied, raw data must first be prepared. Determining which events in a patients’ history should be included or excluded in analysis is a fundamental stage of data analysis which has a significant impact on results, but it is one that is not always described in detail in PM publications. One literature review found that only 22% of papers analysed mentioned data pre-processing (De Roock and Martin, 2022); another observed that 72% of papers either used relatively naive approaches to preprocessing—that is, not considering data quality issues and not relating data pre-processing to their research question—or did not describe it at all (Emamjome et al., 2020).

Some methods for filtering event logs have been proposed. Where there is a high level of variability in possible traces, the most common approach is to filter at the event and the trace level (Marin-Castro and Tello-Leal, 2021). However, *filtering* is often defined in terms of removing noise and logging mistakes (for example, “determin[ing] the likelihood of the occurrence of events or traces based on its surrounding behavior” (Marin-Castro and Tello-Leal, 2021)). Determining whether a recorded event actually happened is a question of interest in healthcare PM, but we argue that there is another step that comes first. Given that any patient will have a long healthcare record, with a large number of possible events, the first question is whether a particular event—regardless of whether it happened or not—is even relevant to the disease or pathway being studied.

An easy and intuitive heuristic that can quickly simplify a large event log is to only examine the most common events in the dataset. However, infrequent events can be very relevant to the process being studied, whilst frequently occurring events can be completely independent of the process. The notion that infrequent behaviour needs to be captured and analysed rather than discarded as noise is a key idea in current thinking around healthcare PM: Munoz-Gama et al. (2022) note that “researchers and practitioners must go beyond simply filtering out infrequent behaviour from the event log”. Tax et al. (2019) demonstrate that filtering activities based on frequency alone does not solve the problems of chaotic activities (that is, events that occur independently of the state of the process), and affects the quality of the final process model.

The second obvious approach is to produce a human-curated list of events to include. This is by far the norm in healthcare research: researchers studying health records typically create a *codelist*, a list of concepts in the dataset’s terminology, which exhaustively defines which exposures and outcomes are being investigated (Williams et al., 2017). The development of such a list is a long process, requiring a clear definition of the clinical feature of interest, a shortlist of the potential codes to include, and an iterative process of expert review (Watson et al., 2017). This process is therefore naturally very time-consuming, and must be repeated for different datasets using different terminologies. However, the advantage is that this approach produces a high-quality codelist, rigorously defined and approved by experts in the field.

Increasingly, attempts have been made to collect these codelists in online repositories, to establish standardised definitions or *phenotypes* for particular diseases and to aid in reproducing research (HDRUK Phenotype Library, n.d.; OpenCodelists, n.d.). However, these libraries are predominantly focused around phenotyping diagnoses rather than procedures, with their codelists largely consisting of diagnostic codes. Given that PM involves a process of curating procedural codes to extract from a healthcare record, which is analogous to the curation of diagnosis to select a cohort, the field of healthcare PM might benefit from an equivalent repository of procedure- and event-centric codelists, to similarly establish common phenotypes of disease pathways and facilitate reproducible research. It has been noted that relatively small variations in diagnosis codelists can lead to noticeable differences in the measured outcomes (Makadia et al., 2023); it therefore follows that that similar changes in a procedural codelist would lead to variations in process models, and greater attention therefore needs to be paid to them.

Within the PM field, a standard approach to generating event logs from healthcare datasets has recently been proposed (Cremerius et al., 2023). In this approach, event filtering involves human curation, with researchers choosing procedures in a terminology based on those described in relevant medical guidelines. However, this approach is limited by the typically vague language of medical guidelines. For example, the UK’s guidance on colorectal cancer simply recommends that “surgery” be offered to patients: this does not specify a particular code or a clear rule for which specific events should be included in a study, and will be interpreted differently by different healthcare systems and individuals (NICE, 2021). Guidelines will also differ by location, making comparing results difficult. There is therefore a need to explore this event selection stage in deeper detail, and establish clear and consistent rules for what is and is not included.

### Ontologies, terminologies, and clinical coding

An automated—or at least semi-automated—codelist curation process has been attempted before; for example, Watson et al.’s framework (2017) selects codes based on whether their text descriptions contain certain search terms. Here, the authors recommend that this step errs on the side of caution, retaining codes unless there exists a clear consensus to reject them. However, clinical datasets typically contain useful semantics already embedded in them, which could be leveraged to assist with this process and provide greater certainty. To implement this, it is important to understand how events are recorded in electronic health records.

In these datasets, events such as diagnoses and procedures are represented by *clinical codes*, alphanumeric identifiers that represent a particular concept in a coding system. In theory, coding systems standardise the reporting of these events, and allow for the conversion of medical records into terms that can be statistically analysed, as well as enabling comparison between hospitals and countries. In practice, healthcare systems encode their data in a wide range of classifications, terminologies, and ontologies that vary between, and even within, countries and healthcare systems (Haendel et al., 2018).

In the United Kingdom, clinical data is largely reported according to two major terminologies:

- **ICD** is an international standard maintained by the World Health Organization. It describes diseases, signs, symptoms, and findings, and is used worldwide to enable comparison of mortality rates and causes. ICD is used to classify diseases and other health conditions within NHS records. Whilst intended for diseases, ICD can also be used to encode procedures: ICD version 9 includes procedures in Volume 3, whilst ICD version 10 does not directly support procedure coding, but the USA maintains a local extension, the Procedure Coding System (ICD-10-PCS) which does.
- **OPCS** is the procedure coding system used by the UK’s National Health Service. OPCS codes consist of one alphabetical character denoting one of 23 “chapters” organised by anatomical site, followed by two numeric digits representing a subcategory, and an optional third digit for further, more specific sub-types.

The OPCS and ICD clinical classification systems are, strictly speaking, *statistical classifications*: a subset of terminologies in which concepts must be mutually exclusive, and arranged mono-hierarchically (every concept having exactly one parent), which aids in statistical reporting (Haendel et al., 2018). However, other standards exist following different structures and for different purposes. The most significant among these is **SNOMED CT**, an international standard containing over 350,000 medical concepts. SNOMED is considered to be an *ontology* because it represents not just a basic hierarchy of concepts, but the complex semantic relationships and polyhierarchies between them. This structure means that entities and relations can be reasoned over using logical rules, and the SNOMED standard includes a language for this exact purpose: the Expression Constraint Language (ECL) (SNOMED International, 2022). This means that logical reasoning over an ontology such as SNOMED can allow us to make logical inferences about healthcare concepts—and potentially, to automatically discover procedure codes that might be relevant to a particular disease or problem.

The idea of using semantics and reasoning to assist in the preparation of codelists has been explored before: Elkheder et al. (2023) demonstrate how reasoning over SNOMED relationships can be used to produce diagnostic codelists, and that these codelists produce similar results to their handcrafted equivalents when used to select patients from a cohort. A natural evolution of this work is therefore to ask whether such methods would also be effective for procedure codes, and whether the resulting process models would resemble each other.

The usage of semantics and ontological reasoning to enhance process mining has also been explored in the past. Alves de Medeiros and van der Aalst (2009) describe *semantic process mining* as the explicit annotation of elements in a log with the concepts they represent, thereby making it possible to automatically reason or infer other relationships between them, and even suggest that such ontologies might be useful in the log cleaning process. Other approaches have used reasoning for abstraction: to group granular events into fewer categories for analysis, and for aggregation: to merge common event sequences into single events based on rules (Leonardi et al., 2019; Remy et al., 2020). Work has also highlighted the apparent gap between between fine-grained events encoded in EHR data, and the more abstract events described in treatment guidelines, with ontology-based abstraction proposed as a way to bridge this gap (Klessascheck et al., 2021).

### Summary

In summary, the preprocessing stages are a key part of the process mining framework. Modeling assumptions and decisions, such as which events in a raw health record are included and which are discarded, will inevitably have an impact on the outcome of any analysis. Current practice has significant flaws: filtering an event log based on the frequency of events risks losing useful information, and hand-curating codelists is, whilst reliable, exceptionally time-consuming, and leads to different results in different studies. Exact codelists and preprocessing steps used in studies are not always elaborated upon in detail in publications, which limits the ability of researchers to reproduce results and compare studies. However, the fact that EHR data is encoded according to standards and ontologies means that it may be possible to use logical reasoning in the log preparation stages.

Whilst progress has been made towards standardising event log preparation in healthcare PM, there is a need to explore the event selection stage in deeper detail, and to establish clear and consistent rules for what is and is not included. This paper describes one such potential approach, using ontologies to derive a rationale for the inclusion of individual events.

## Methods

This section describes the proposed approach for ontology-informed event extraction, and further describes the methods used for data transformation and evaluation.

**Fig. 1:**
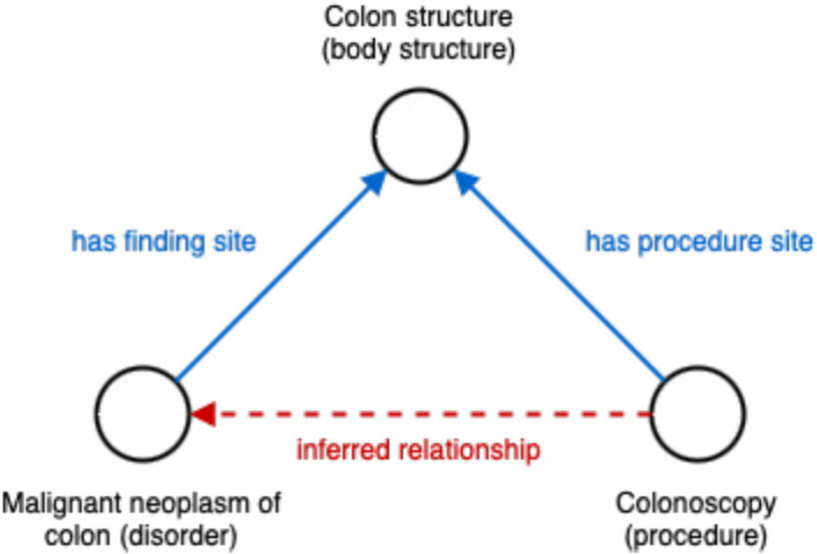
Inferring a new relationship between two entities from two known relationships

### Event extraction

Firstly, for PM to be effective, a raw health record needs to be filtered down to only include the events relevant to a particular research question. The approach proposed here uses SNOMED ECL queries to automatically identify procedures relevant to a particular disease area. Four candidate SNOMED ECL queries for identifying concepts of interest were developed based on preliminary investigations.

The basic intuition behind this method is simple: given a procedure *P*, an anatomical site *S* and a disease *D*, if the relationships $\left( P, \textit{ procedure site}, S \right)$ and $\left( D, \textit{ finding site}, S \right)$ exist in the ontology, it can be inferred that *P* is a relevant procedure to *D* (Fig. 1). It is not guaranteed to be an acceptable or recommended treatment, but this logical link means that it is at least a plausible one. We begin the search with a specified diagnosis, rather than another concept such as a site, because this is the level at which research questions are typically focused (e.g. “what do treatment pathways for colorectal cancer look like?”).

This logic can be formulated in ECL as the following query:
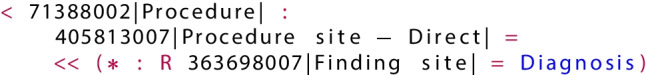


In this notation, the < operator retieves all concepts that are a descendant of another, $<<$ retrieves concept that are either a descendant of another or the concept itself, $*$ is a wildcard, indicating any concept,  :  indicates refinement, and *R* indicates a reversal - i.e. a retrieval of the set of attribute values that exist given a particular attribute and a set of concepts. 71388002|Procedure| represents the SNOMED concept *procedure*, which has the ID 71388002. Thus, this command retrieves, from the set of all descendants of *procedure*, concepts whose attribute *procedure site* is equal to, or descended from, any concept within the set of possible *finding sites* for the given *diagnosis*. A wide range of further constraints are possible: based on the results of this basic query, the exact formulation is improved and iterated upon.

### Data transformation

Once a set of relevant SNOMED concepts has been retrieved, they need to be converted into the terminology used by retrospective datasets, and into a readable and useful form.

#### Mapping between code systems

In previous work (Dwyer et al., 2024), we used the National Health Service’s official SNOMED to OPCS mapping files to convert the output of the SNOMED ECL queries to a codelist compatible with the OPCS and ICD coded EHR data; these mapping files came with some downsides. These maps are intended to assist clinical coders in converting codes for billing purposes. Most SNOMED concepts map to either a choice of multiple OPCS codes, or combinations of codes, with the expectation that clinical coders make the choice. This is acceptable for filtering purposes, since the main aim is to establish plausibility (“could this event be related to this disease?”) rather than to guarantee causality (“this event must have been intented as a treatment for this disease”). Given the precedent that questionable codes should be retained unless there is a clear rationale for exclusion (Watson et al., 2017), we assumed that any OPCS code that is mapped in any way to a SNOMED concept could plausibly be related to it. However, this method led to relatively low query precision, and meant that extra maps were required to translate to ICD-9 Vol. 3 and ICD-10-PCS codes, in which we identified some obvious gaps.

To address these issues, this experiment uses a different approach: we map SNOMED concepts to the OPCS and ICD systems using the OMOP Common Data Model (Reich et al., 2024). Previous research has shown that UK format EHR data can be converted to this data model, with 99% of OPCS concepts being covered (Papez et al., 2021, 2023).

#### Code abstraction

A major issue with healthcare PM is the complexity of the process models created, in particular the propensity for so-called “spaghetti models”. There are over 10,000 possible events in the OPCS code system; even after filtering to just the relevant ones, we can still be left with a very large number, creating a need to group them together into meaningful and interpretable categories. Whilst there are several ways to achieve this, this experiment used the code’s text descriptions to identify natural groups. A set of keywords was established based on the groups used in the colon and lung cancer lists, and these were used to group codes into categories. For example, any concept that contained ‘resection’, ‘excision’, ‘lesion’ or ‘exentoration’ in its description was mapped to ‘resection’.

### Evaluation

Three approaches are used to evaluate the quality of results from the ECL queries: a comparison of the output codelist against established research codelists, a comparison of the resulting process models against models created from benchmark codelists, and a comparison of the codelists against the statistical distribution of codes in the data.

**Table 1:** List of benchmark codelists used in this study and their sources

	Disease	*n*	Source
A	Appendicitis	14	HDRUK (PH106 / 212) (HDRUK Phenotype Library, n.d.)
B	Cataract	32	HDRUK (PH131 / 262) (HDRUK Phenotype Library, n.d.)
C	Glaucoma	21	HDRUK (PH176 / 352) (HDRUK Phenotype Library, n.d.)
D	Lung cancer	76	Consultation with domain experts
E	Colorectal cancer	497	CORECT-R (CORECT-R Data Coding v1.0, 2020) + consultation with domain experts

IDs and version numbers are also provided for codelists from the HDRUK library

#### Comparing codelists

Firstly, we compare each output codelist against established codelists from medical research studies. To ensure that we gain a full picture of our methods’ effectiveness, and how that effectiveness varies by disease, we use codelists from three different sources, and of different sizes (Table 1).

Codelists A, B, and C, which represent appendicits, cataract, and glaucoma, come from the HDRUK Phenotype Library. These were chosen from the relatively small number of available codelists which contain procedural codes, and priority was given to longer codelists in order to produce more meaningful precision and recall statistics (for example, diverticular disease provides only one OPCS code, which would make evaluation meaningless). These three codelists all originate from the same publication (Kuan et al., 2019), in which the presence of particular OPCS codes (in addition to or in place of other diagnostic and procedural codes) are used to determine whether a patient has or does not have a particular condition.

Codelist D was constructed based on lung cancer procedures from consultation with domain experts. Codelist E comes from an established research database, the COloRECTal cancer Repository (CORECT-R) (Downing et al., 2021). This project has published an extensive codelist of 192 different OPCS codes (CORECT-R Data Coding v1.0, 2020), divided into five treatment categories, that cover the main treatments for any colon or rectal cancer. We used a previously produced extension of this codelist, which adds several additional codes for relevant diagnostic tests, chemotherapy, radiotherapy, and surgical procedures to gain a full picture of the entire patient journey for colorectal cancer.

These codelists come from different sources and serve different purposes: A, B, and C act as minimum thresholds regarding whether or not a patient should be considered to have a particular disease, whilst D and E are closer to exhaustive lists detailing all possible procedures for a disease, in order to aid researchers studying the specific treatment processes. It is likely that this second set of codelists are designed to requirements more closely resembling those needed for PM; this range of different sources is deliberately included to consider how well this approach generalises to different scenarios. The output of each ECL query is evaluated according to precision (the proportion of retrieved codes that were in the reference list) and recall (the proportion of codes in the reference list successfully retrieved).

#### Comparing processes

To investigate the extent to which our methods change the results of analyses, we use the codelists produced by our queries to generate directly-follows graphs of treatment processes from real-world datasets. We use data collected by Oxford University Hospitals (OUH) NHS Foundation Trust, which operates four hospitals in Oxfordshire, England. From the raw health records of patients diagnosed with either lung (ICD-10 codes C33*, C34*) or colon (C18*) cancer, we produce two directly-follows graphs of events for each disease, one filtered from a benchmark OPCS codelist, and one filtered according to an OPCS codelist derived from our SNOMED ECL queries.

#### Comparing statistics

A final useful method of evaluating our approach is to compare it against a statistical approach, in which the relevance of events is determined based on their correlation with a particular diagnosis. We can compare the set of correlated events with the events in a logically constructed codelist, and identify codes that have no clear logical link to the target disease but are still associated with a diagnosis according to the data.

However, a limitation of the OUH dataset is that the data available only consists of patients with colorectal or lung cancer, making it impossible to see which codes are over-represented in the colon and lung cohorts compared to the general population. Therefore, we also make use of MIMIC-IV (Johnson et al., 2020, 2023), a freely accessible EHR dataset from Massachusetts, USA which has been widely used in previous PM studies. In MIMIC, each procedure is characterised by an ICD code, using a mixture of ICD versions 9 and 10, which can be converted to and from SNOMED using the same OMOP CDM approach used for OPCS.

The colon and lung cancer patients in MIMIC were divided into a case cohort of patients with the target diagnosis and a control cohort of those without. Through statistical testing with Fisher’s exact test, we identify codes with a statistically significant difference in frequency between the two cohorts, and therefore some association with a diagnosis of colon or lung cancer.

## Results

In this section, we present the initial results of our proposed approach by comparing the quality of the generated codelists against our reference lists, by comparing the process models generated using these lists, and by examining the distribution of codes in a supplementary dataset.

### Query development

Initially, concepts were retrieved based on the query outlined in Section 3.1 (referred to here as **Query 1**):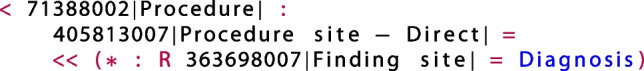 During initial tests, this query retrieved many codes which were only tangentially relevant: applying this example to colon cancer, for example, returned a number of codes describing *oesophagectomy*, the surgical removal of the oesophagus. Because this procedure sometimes involves replacing the oesphagus with material from the colon, several concepts therefore include the colon as a procedure site. In practice, this procedure is very unlikely to be related to colon cancer; the oesophagus is the primary site of this procedure, and the colon is of secondary concern. Therefore, to exclude such concepts we introduced an additional cardinality constraint that procedures must have one and only one site (**Query 2**): 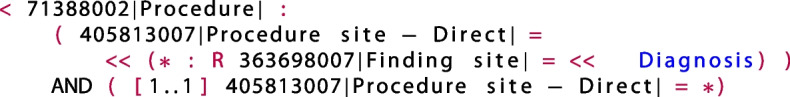


A second issue with Query 1 was that it also retrieved some procedures that were seemingly totally irrelevant. For example, in some older releases of SNOMED CT, the concept representing *malignant neoplasm of colon* had child concepts including *malignant neoplasm of rectosigmoid junction metastatic to brain*, which meant that the brain was also automatically introduced as a procedure site of interest, so any number of codes relating to the brain are returned as a result. In case such examples exist within other diseases, we created a more specific query: instead of requesting any possible site for any child concept of the target disease, we request only procedures that involve a specific, named anatomical concept (**Query 3**): 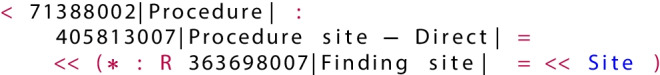 Finally, we combined the new rules introduced in Queries 2 and 3 to constrain our output to those procedures with exactly one procedure site, *and* a procedure site that is a specific named site, or a descendant thereof (**Query 4**): 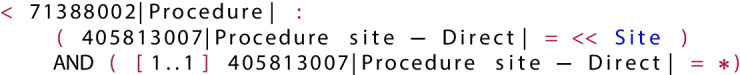 When running these queries on cancer diagnoses, there also exist several other procedures that are highly relevant but not site specific: for this reason, on these queries we append an additional request for chemotherapy and radiotherapy codes: 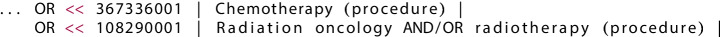

**Fig. 2:**
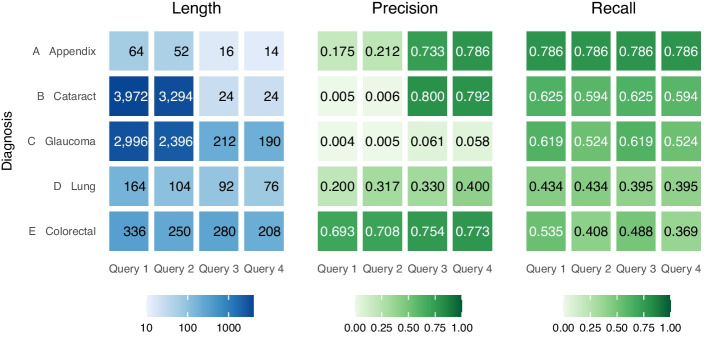
Length (i.e. number of codes), precision and recall of codelists resulting from query formats 1-4 against five different target diagnoses

### Comparing codelists

Figure 2 compares the results of Queries 1 through 4 in five different disease scenarios. The queries were run against the February 2024 release of SNOMED CT UK Edition. OPCS codes in chapters Y and Z—supplementary codes that describe additional information such as sites or approaches—are excluded from calculations since as a rule they never appear in the primary position in records, and therefore have little bearing on PM results.

In terms of raw codelist size, moving from the more general Query 1 to the more restrictive Query 4 consistently reduces the number of codes returned, as expected. However, two diagnoses—cataract and glaucoma— produced exceptionally large result sets for Queries 1 and 2. This occurs due to the existence of concepts in SNOMED that represent multiple diagnoses, for example the concept *glaucoma and sleep apnea* means that both *structure of the eye* and *structure of the respiratory system* are both introduced as acceptable procedure sites, so all relevant procedures are added accordingly. This is significantly improved by the introduction of the more narrow site definition in queries 3 and 4.

Precision was generally low. This is most visible again in cataract and glaucoma, where the exceptionally large codelist size made meant that inevitably a very small number of those codes would be relevant. However, it is also noticeable that in the case of the cataract codelist, and to a lesser extent in appendicitis, Queries 3 and 4 represented a significant jump in precision, suggesting that the move from a specific disease to a single named procedure site eliminated a large number of irrelevant concepts. Colon cancer is the notable outlier in terms of precision, likely owing to the significantly larger size of the benchmark codelist compared to the others.

Recall, by contrast, was relatively high across all scenarios tested. It was entirely constant across all queries for appendicitis, indicating that the changes to constraints did not affect any of the targeted codes. In every other case, recall was lower in Query 4 than in 1, indicating that increasing constraints—while effective at reducing irrelevant codes, as evidenced by the levels of precision—also removed some relevant codes.

**Fig. 3:**
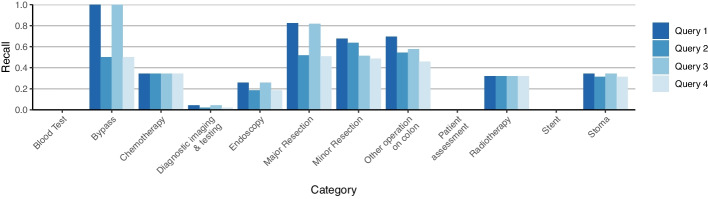
Recall of queries by category compared to the extended CORECT reference set

Figure 3 shows the recall of each colon cancer query, further broken down by the target concepts’ categories according to the benchmark codelist. Concepts representing blood tests, diagnostic imaging and testing and patient assessment were never retrieved, presumably because they are not disease specific and therefore do not have a clear site. Similarly, endoscopies of the upper gastrointestinal tract were never retrieved as they do not have a relevant procedure site, but such procedures are commonly included in colon cancer analyses as they commonly form part of the route to diagnosis. Recall was highest in surgical topics, since they were well annotated with clear procedure sites in the ontology. The differences in constraints between queries 1-4 only had a noticeable affect on these codes.

**Fig. 4:**
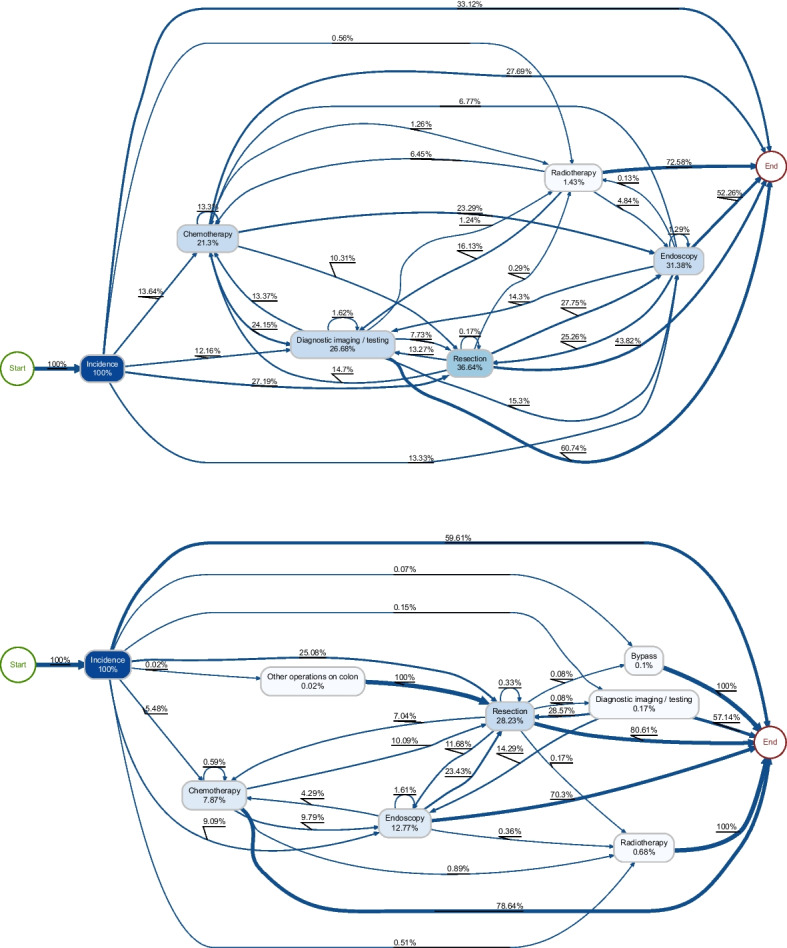
Directly-follows graphs for colon cancer benchmark codelist (top) and Query 1 (bottom)

### Comparing processes

**Fig. 5:**
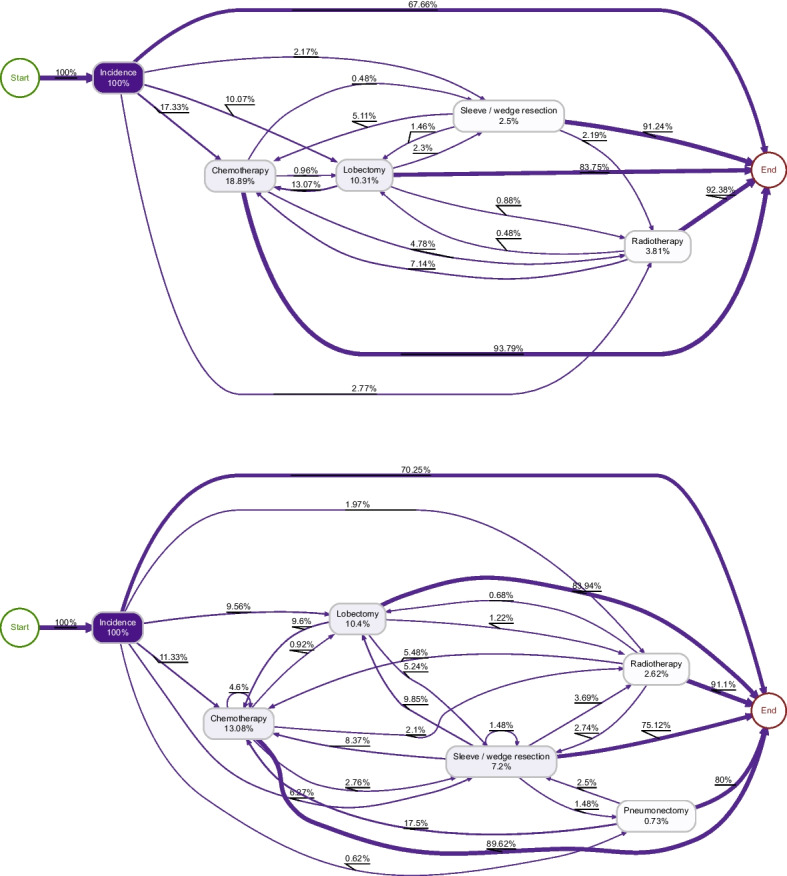
Directly-follows graphs for lung cancer benchmark codelist (top) and Query 1 (bottom)

To investigate the usefulness of these codelists for PM, we generated directly-follows graphs for colon and lung cancer patients from the OUH dataset, based on the generated codelist according to Query 1 (chosen for consistently displaying the highest recall) and the known benchmark codelist. In these graphs (Figs. 4 and 5), node labels represent relative case frequency, i.e. the number of patients experiencing that event, whilst edge labels represent the relative antecedent frequency, i.e. the proportion of instances of the source event that are followed by the destination event.

#### Colon cancer

For clarity and simplicity, *minor* and *major* resection were grouped into one *resection* category, as were *lower GI* and *upper GI endoscopy*. Activities that only occured in a very small proportion of patients (stoma, stent, bypass, other, imaging, assessment) were excluded.

The two processes resemble each other in many ways. A similar proportion of patients underwent resections (28.23% in our codelist, 36.64% according to the benchmark codelist), reflecting the reasonably high recall in these topics. Both processes share similarities: the most common first event was resection; imaging and radiotherapy were typically the last event in a pathway; and radiotherapy very rarely follows a resection. However, there are also some significant differences: the proportion of patients experiencing chemotherapy, imaging and endoscopies were all markedly lower in our model than the CORECT model. Additionally, 59.61% of patients did not experience any events of interest, compared to 33.12% according to the benchmark list.

#### Lung cancer

In lung cancer, there was a higher level of agreement between the two process models. In particular, a very similar rate of lobectomies were recorded by both models. Chemotherapy and radiotherapy rates were also similar. The smaller scale of discrepencies between these two models reflects the fact that the number of possible codes for lung cancer are considerably fewer than for colon cancer, given that treatment via surgery is less common.

**Table 2:** Procedure codes most strongly associated with a colon cancer diagnosis in the MIMIC dataset that did not also appear in our derived codelists

ICD concept		Prevalence (%)		Odds ratio	*p*
Code	Description	Control	Case		
0DTF4ZZ	Resection of Right Large Intestine, Percutaneous Endoscopic Approach	0.05	6.69	137.57	7.88*e*-108
0DTG4ZZ	Resection of Left Large Intestine, Percutaneous Endoscopic Approach	0.01	1.15	82.53	6.76*e*-18
4593	Other small-to-large intestinal anastomosis	0.15	7.65	55.54	1.39*e*-99
0DTF0ZZ	Resection of Right Large Intestine, Open Approach	0.07	3.25	51.38	1.41*e*-42
4594	Large-to-large intestinal anastomosis	0.08	2.87	34.97	1.27*e*-33
4686	Endoscopic insertion of colonic stent(s)	0.05	1.53	31.29	5.14*e*-18
0DJD4ZZ	Inspection of Lower Intestinal Tract, Percutaneous Endoscopic Approach	0.06	1.15	20.93	4.51*e*-12
4579	Other and unspecified partial excision of large intestine	0.06	1.05	16.46	3.49*e*-10
0D1B0Z4	Bypass Ileum to Cutaneous, Open Approach	0.18	2.58	15.08	6.71*e*-22
4620	Ileostomy, not otherwise specified	0.12	1.43	12.46	8.89*e*-12

**Table 3:** Procedure codes most strongly associated with a lung cancer diagnosis in the MIMIC dataset that did not also appear in our derived codelists

ICD concept		Prevalence (%)		Odds ratio	*p*
Code	Description	Control	Case		
07T70ZZ	Resection of Thorax Lymphatic, Open Approach	0.00	0.90	361.96	5.75*e*-43
07B70ZZ	Excision of Thorax Lymphatic, Open Approach	0.01	1.12	122.81	1.32*e*-47
07B70ZX	Excision of Thorax Lymphatic, Open Approach, Diagnostic	0.01	1.53	115.38	1.02*e*-63
07B74ZX	Excision of Thorax Lymphatic, Percutaneous Endoscopic Approach, Diagnostic	0.07	4.74	66.48	6.81*e*-176
3228	Endoscopic excision or destruction of lesion or tissue of lung	0.03	1.90	65.91	1.60*e*-71
3422	Mediastinoscopy	0.05	2.68	59.59	5.34*e*-98
3230	Thoracoscopic segmental resection of lung	0.02	1.12	56.28	1.96*e*-41
07D78ZX	Extraction of Thorax Lymphatic, Via Natural or Artificial Opening Endoscopic, Diagnostic	0.03	1.25	50.09	1.17*e*-44
07T74ZZ	Resection of Thorax Lymphatic, Percutaneous Endoscopic Approach	0.02	1.15	49.59	2.50*e*-41
07B74ZZ	Excision of Thorax Lymphatic, Percutaneous Endoscopic Approach	0.04	1.56	44.86	6.08*e*-54

### Comparing with statistics

The procedure codes with the strongest association with a colon or lung cancer diagnosis, according to the MIMIC dataset, are shown in Tables 2 and 3 respectively. Each code was counted in the diagnosed and non-diagnosed population, and the rate of occurance compared by calculating an odds ratio and statistical testing. Listed in these tables are those codes that were strongly associated with a colon or lung cancer diagnosis—that is, they appeared $>10$ times in the case cohort and their association was statistically significant ($p<0.05$) according to Fisher’s exact test—and did not appear in the codelist derived from Query 1.

For colon cancer, most of the “missing codes” related to the large intestine, and therefore were not found by our queries as they were not tagged with a descendant of “colon” as a procedure site (“colon” is itself a descendant of “large intestine” in SNOMED). The exception here is *endoscopic insertion of colonic stent(s)*, which does not have a recorded map to a particular SNOMED code in the OMOP data model, although it does have an *is a* relation to a similar SNOMED concept. Lung cancer follows a similar pattern, with many of the codes relating to the lymphatic system and therefore not having a relevant procedure site tagged. *Endoscopic excision...* again did not have a map to a directly equivalent SNOMED concept.

## Discussion

These results suggest that the proposed approach to extracting event logs using queries over ontologies is capable of approximating an expert-prepared codelist with some accuracy. There is a clear trade-off between precision and recall: depending on the query structure, the results lie on a spectrum between a relatively complete codelist with a large number of extra codes, or an incomplete codelist in which every code is likely be relevant.

Performance varies depending on the type of event: surgeries are often easy to identify, owing to their clear structure and the prevalence of useful annotations in the SNOMED ontology, whereas relevant but non-disease specific events such as chemotherapy, diagnostic tests and imaging need to be explicitly specified. In some cases, there exist relevant concepts that don’t have a clear ontological relationship to known concepts: procedures in adjacent locations can happen due to surgical complications or cancer metastasis. However, any widening of definitions needs to be balanced with the risk of increasing the number of irrelevant codes. Query constraints always need to be guided by the research question and purpose.

Performance also depends on the choice of, and the original purpose of, the benchmark codelist. Codelists A, B, and C were defined by their original authors for the purpose of selecting a patient cohort, rather than to explicitly study the care processes of those patients, which is the purpose of Codelists D and E. Codelists are not designed with specific intents in mind, and it is important for PM researchers to bear this in mind when reusing them for comparison.

The supplementary analysis of the the MIMIC dataset indicated that there are still many concepts that are clearly correlated with a particular diagnosis, but have no obvious links according to ontologies. This suggests that a key area for future work is better integration between logical and statistical approaches, to leverage the benefits of both.

Our results also highlighted that abstracting events into meaningful and useful categories is still a key challenge. When developing our methodology, three approaches were considered. One option was using the structure of the data’s original terminology. OPCS codes consist of four characters, which describes three hierarchical levels. For example, the code H01.1 represents *Emergency excision of abnormal appendix and drainage however further qualified*, H01.2 represents *emergency excision of abnormal appendix not elsewhere classified*, and H01.3 represents *emergency excision of normal appendix*. Collectively, all of these codes fall under the chapter H01 *emergency excision of appendix*, which itself falls under Chapter H, *lower digestive tract*. Therefore, it is possible to aggregate codes by grouping them according to these chapters and subchapters. Aggregating codes using this structure was in practice challenging. Since OPCS chapters are arranged at the highest level by site (with the exception of a *diagnostic imaging, testing, and rehabilitation* and a *miscellaneous* chapter), almost every code related to a particular disease will come from one chapter, resulting in almost all events being aggregated together. For example, Query 1 applied to colon cancer yielded 257 codes from the H (“lower digestive system”) chapter, 24 codes from the X (“miscellaneous operations”) chapter, and fewer than 20 codes from every other chapter. Moving down a level to three-character OPCS codes creates the opposite problem: there are far too many subcategories for this information to be useful for event aggregation. In the example of Query 1 for colon cancer, the results span 80 three-character concepts, each containing at most 10 codes, which is far too many to create an interpretable process model. Therefore, the level of abstraction means that the OPCS terminology structure in and of itself is not a useful framework for event aggregation.

A second approach is to aggregate the codes before their transformation, within the SNOMED terminology. SNOMED concepts are semantically annotated with additional relations, for example procedures can have *method* or *approach* relationships. These relationships can therefore be used to infer group relationships, for example by categorising any concept with method *imaging* as imaging, or anything with methods *repair* or *excision* as surgery. By examining the most common *methods* relationships that exist within the codelist, we hand wrote a set of maps that take advantage of this structured knowledge. In this case, the main barrier to useful aggregation was the fact that many OPCS concepts were mapped to multiple possible categories in the SNOMED terminology. In most cases this was because the source SNOMED concept had multiple *method* relationships, but occasionally because one OPCS concept could be mapped to multiple SNOMED concepts. Additionally, the high number of possible *methods* created a very large number of categories; in the case of Query 1 for colon cancer, there were 48.

A major issue shared by both the OPCS and SNOMED-level aggregation approaches is that the categories do not clearly align with those from the benchmark codelists. This means that any resulting process models are not comparable with those from a different source codelist. For this reason, we utilised the text descriptions of each code, defining a set of categories equivalent to those in the colon and lung cancer benchmark lists, and matching codes to these based on a keyword search. These keywords were chosen based on patterns identified from manually examining the benchmark codelist, to ensure as close an alignment as possible. This is the most manual and intensive approach, and less intuitive since it does not rely on existing ontological structure, but has the key advantage that it can be specified and developed by researchers according to their needs, and categories can be designed to match existing categorisations from comparable codelists.

It is therefore clear that at present, the structure of the key ontologies used in healthcare data do not easily support aggregation into a useful level, and some level of human curation is realistically still required. Effective abstraction for research also depends on the research question: modelling an overarching treatment process requires a different abstraction level to trying to determine smaller process differences within a particular treatment pathway, for example.

The proposed approach is capable of providing a reasonably accurate list on which to begin domain expert-lead discussions, speeding up the initial stages of the development process. It allows the majority of concepts in a codelist to be described through as a set of intuitive and explainable inclusion criteria in a standard language. As a result, the codelists themselves consist of concepts from a standard ontology that can then be translated into the languages used by individual datasets—although this is highly dependent on both the completeness of the original ontology and the quality of mappings available. This approach could also be informative in situations where very small amounts of data are available, limiting the usefulness of statistical analysis of the most common codes. Finally, the process of specifying a set of requirements for desired events in terms of their relationship to a particular target disease keeps the PM method focused on a concrete health issue, ensuring clinical meaningfulness and good alignment between PM studies and current research questions in healthcare.

We have previously discussed aligning PM practice with methods used in epidemiology and healthcare research, to improve the usefulness and relevance of PM methods to the healthcare community (Chammas et al., 2024). In this paper, we have furthered this goal by taking codelist curation, a common process in the medical literature, and considered its relevance to process mining and how it might be enhanced by structured domain knowledge. Domain knowledge, even in the form of well-established and curated ontologies, is rarely perfect and cannot instantly automate the process of event log preparation. However, it can provide a meaningful framework to generate ideas to be iterated upon, and inspire conversations with domain experts.

## Conclusions

In this work we considered how to support healthcare organisations in extraction and analysis of large-scale healthcare processes. Our main contributions are:

- an approach to event extraction in electronic health record data using knowledge from ontologies. Sets of logical constraints can be defined that will produce lists of ontological concepts, and these lists can approximate those that would be prepared by domain experts.
- an application and demonstration of these techniques in a widely used ontology and on real patient data. We successfully extracted process models from real data based on our logically constructed codelists, and established the areas in which they do and do not resemble those constructed from other means.
- an investigation into the use of the same ontological structure for event aggregation. We identified limitations in the specific ontologies used in this work, and concluded that human aggregation is necessary and even desirable to ensure that results remain aligned and comparable with previous and future research.

The approach is far from a replacement for discussion and input from domain experts; it in fact underlines the importance of a deep understanding of the subject area, of the research question, and of the very nature of electronic health record data. It emphasises the importance of approaches to process mining that are informed by domain expertise, but that are also enhanced and expedited by the use of structured ontological knowledge and statistics.

This study highlights that no single basic approach—whether reliant on ontologies, on conversation with domain experts, or on pure statistics—can fully capture the complex nature of healthcare processes; a well-rounded approach encompassing all three perspectives is required to deeply explore health processes and to answer questions around modern healthcare delivery.

## Supplementary information

We provide the results of all of our queries, along with our benchmark codelists, in an accompanying file.

## Supplementary Information

Below is the link to the electronic supplementary material.

Supplementary file 1 (xlsx 1571 KB)

## Data Availability

OUH patient data is not publicly available due to ethical and legal restrictions. The MIMIC dataset is publicly available (Johnson et al., 2020). All query and benchmark codelists are available in an accompanying file.
